# Genes with spiralian-specific protein motifs are expressed in spiralian ciliary bands

**DOI:** 10.1038/s41467-020-17780-7

**Published:** 2020-08-20

**Authors:** Longjun Wu, Laurel S. Hiebert, Marleen Klann, Yale Passamaneck, Benjamin R. Bastin, Stephan Q. Schneider, Mark Q. Martindale, Elaine C. Seaver, Svetlana A. Maslakova, J. David Lambert

**Affiliations:** 1 Department of Biology, University of Rochester, Rochester, NY 14627 USA; 2 Oregon Institute of Marine Biology, University of Oregon, Charleston, OR 97420 USA; 3 Whitney Laboratory for Marine Bioscience, University of Florida, 9505 Ocean Shore Blvd., St. Augustine, FL 32080 USA; 4 Kewalo Marine Laboratory, PBRC, University of Hawaii, 41 Ahui Street, Honolulu, HI 96813 USA; 5 Department of Genetics, Development and Cell Biology, Iowa State University, 503 Science Hall II, Ames, IA 50011 USA; 6 Present Address: Department of Ecology and Evolutionary Biology, Yale University, New Haven, CT 06520 USA; 7 Present Address: Molecular, Cellular and Developmental Biology, University of California, Santa Barbara, Life Sciences Building, Santa Barbara, CA 93106 USA; 8 Present Address: School of Life Sciences, University of Sussex, Brighton, BN1 9RH UK; 9 Present Address: Institute of Cellular and Organismic Biology, Academia Sinica, Taipei, Taiwan

**Keywords:** Embryology, Evolutionary developmental biology

## Abstract

Spiralia is a large, ancient and diverse clade of animals, with a conserved early developmental program but diverse larval and adult morphologies. One trait shared by many spiralians is the presence of ciliary bands used for locomotion and feeding. To learn more about spiralian-specific traits we have examined the expression of 20 genes with protein motifs that are strongly conserved within the Spiralia, but not detectable outside of it. Here, we show that two of these are specifically expressed in the main ciliary band of the mollusc *Tritia* (also known as *Ilyanassa*). Their expression patterns in representative species from five more spiralian phyla—the annelids, nemerteans, phoronids, brachiopods and rotifers—show that at least one of these, *lophotrochin*, has a conserved and specific role in particular ciliated structures, most consistently in ciliary bands. These results highlight the potential importance of lineage-specific genes or protein motifs for understanding traits shared across ancient lineages.

## Abstract (web summary)

Spiralians have ciliary bands, used for locomotion and feeding, but defining molecular features of these structures are unknown. Here, the authors report a gene, *Lophotrochin*, that contains a protein domain only found in spiralians, and specifically expressed in diverse ciliary bands across the group, which provides a molecular signature for these structures.

## Introduction

**Fig. 1:**
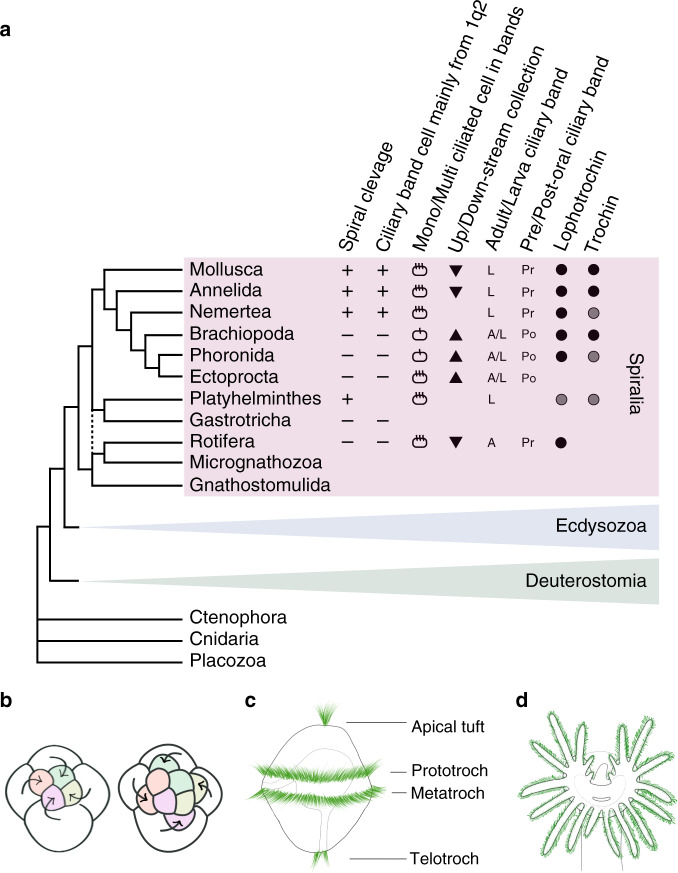
The clade Spiralia and spiralian development. **a** Phylogeny of the Spiralia within the Metazoa, with key characters indicated, focusing on those related to the ciliary bands^2–6^. The characters describe the generalized differences among groups, but not necessarily true for all the members within the group. The prototroch of molluscs, annelids, and basal nemerteans is derived from the vegetal daughters of the first quartet of micromeres (1q^2^), supporting homology. This is not true for other spiralians; in most groups spiral cleavage has been lost, so it is not possible to identify the equivalent cells, even if fate-mapping studies have been performed. Platyhelminth flatworms do have spiral cleavage but the contribution of their 1q^2^ cells has not been specifically determined, so cell lineage does not support the homology of flatworm larvae ciliary bands with molluscs, annelids and nemerteans. Cell symbol with one line: monociliated cell in bands; cell symbol with multiple lines: multiciliated cell in bands. Triangle orientation indicates whether the ciliary bands in the group collect food particles upstream or downstream^23^. L: larva ciliary band; A/L: adult and larva ciliary band Pr: preoral ciliary band; Po: post-oral ciliary band. Black dots indicate where the gene was recovered and in situ performed; gray dots indicate where the gene was recovered in the phylum but we were unable to perform the in situ hybridization. No dot shown indicates the gene was not recovered; in the case of Rotifera, the gene was not recovered in its genome, in all other cases, no genome is available for the search. **b** Spiral cleavage, characterized by the highly stereotypical angles of the cell divisions, and the characteristic asymmetries in the size of daughter cells. Left panel shows orientation of cell cleavages that give rise to the first set of micromere daughters at the eight-cell stage, and the right panel shows the birth of the second set of micromeres. **c** A generalized trochophore larva, prototroch and other ciliated structures in green. **d** Lophophore of an adult lophophorate, cilia in green.

The large multiphylum group Spiralia contains about 11 of the approximately 25 phyla of bilaterian animals; a subset of it is known as the Lophotrochozoa (see Fig. 1a^1–6^). The group includes molluscs, annelids *sensu lato* brachiopods, phoronids, nemerteans, bryozoans, platyhelminth flatworms, and rotifers. The Spiralia arose shortly after the origin of bilaterians, probably at the beginning of the Cambrian^7,8^, and constitute one of the three major ancient bilaterian lineages along with ecdysozoans and deuterostomes.

The adult body plans of spiralians are extremely diverse, but several traits are conserved among some spiralian phyla. Molluscs, annelids, nermerteans and polyclad flatworms share a conserved pattern of early development called spiral cleavage (Fig. 1b; reviewed in refs. ^9,10^). Prominent ciliary bands used for locomotion and/or feeding are broadly distributed across spiralian phyla but their homology within the group is controversial^11–20^. There are striking similarities in the structure and functional roles of larval ciliary bands among some groups: representatives of both annelids and molluscs share the distinctive trochophore larval type (Fig. 1c). The trochophore’s main ciliary band is the prototroch, which is composed of cells with multiple large cilia that propel the larva and/or capture food with a downstream flow pattern^21^. In molluscs and annelids, the prototroch originates from the same cells of the early embryo (1q^2^, or the vegetal daughters of the 1st quartet of micromeres), and is widely considered homologous among these trochophore larvae^13,14,17,22^. However, the homology is less clear for the other ciliary bands of trochophore larvae, such as the metatroch and telotroch^11,12,15,17–19,23^. Other spiralian groups that lack typical trochophore larvae often have prominent ciliated bands in larvae, adults or both, but their homology is uncertain; there is considerable variation in their structure and function and they do not derive from cells that are clearly homologous to 1q^2^, as in molluscs, annelids and nemerteans (summarized in Fig. 1; reviewed in refs. ^14,17–20^; discussed below). Furthermore, ciliary bands are found in some groups outside the Spiralia, like the echinoderms and the ctenophores, underscoring how they may evolve convergently (metazoan ciliary bands are reviewed in ref. ^24^). In this context, any marker that unites ciliary bands in one clade might be significant.

Here we report a known molecular commonality between diverse spiralian ciliary bands; these came from a screen for genes that are specific to spiralians. Lineage-specific genes are conserved within one lineage but absent in outgroups^25–27^. These genes are a potential source of evolutionary novelties, but the biological functions of lineage-specific sequences and their roles in evolution are largely unknown, especially for understudied lineages such as the Spiralia^27–29^. In general, to be considered a novel gene, a gene should arise at a new chromosomal locus, either by segmental duplication, transposition, or de novo origin from previously non-coding sequence^27,30–33^. These modes have mostly been demonstrated in recently diverged species groups; rigorously demonstrating gene origin in an ancient lineage is very problematic because of genomic rearrangements, gene loss, and the loss of intermediate taxa. Thus, screens for ancient lineage-specific genes are based on homology-based methods, and the precise mode of origin generally remains unknown. Here we carry out a screen for genes that are restricted to the Spiralia, because we wonder whether these might be involved with spiralian-specific traits. In a survey of representatives of five spiralian phyla, we find two such genes that are largely restricted to ciliary bands across the group. These results highlight the potential importance of lineage-specific genes or protein motifs for understanding phenotypic evolution.

## Results

### *Lophotrochin* and *trochin* are expressed in the primary ciliary band of *Tritia*

To find genes that have arisen de novo or evolved new functions in the spiralian common ancestor, we carried out a bioinformatic screen for protein sequences that are strongly conserved within the Spiralia, but are not recognizable or poorly conserved in other organisms. Briefly, we screened for genes that were detectable at a BLAST e value below 10e-7 in at least one genome of molluscs, annelids and platyhelminthes, respectively, but not present in any non-spiralian outgroup genome, or the NCBI nr database at an e value below 10e-5 (see Methods for details). This approach differs from other screens that find new lineage-specific paralogs, such as homeobox gene paralogous groups that are restricted to a particular lineage (e.g., ref. ^34^). By definition, the protein motifs recovered in our screen have no detectable sequence similarity that can assign them to protein families outside of spiralians.

**Fig. 2:**
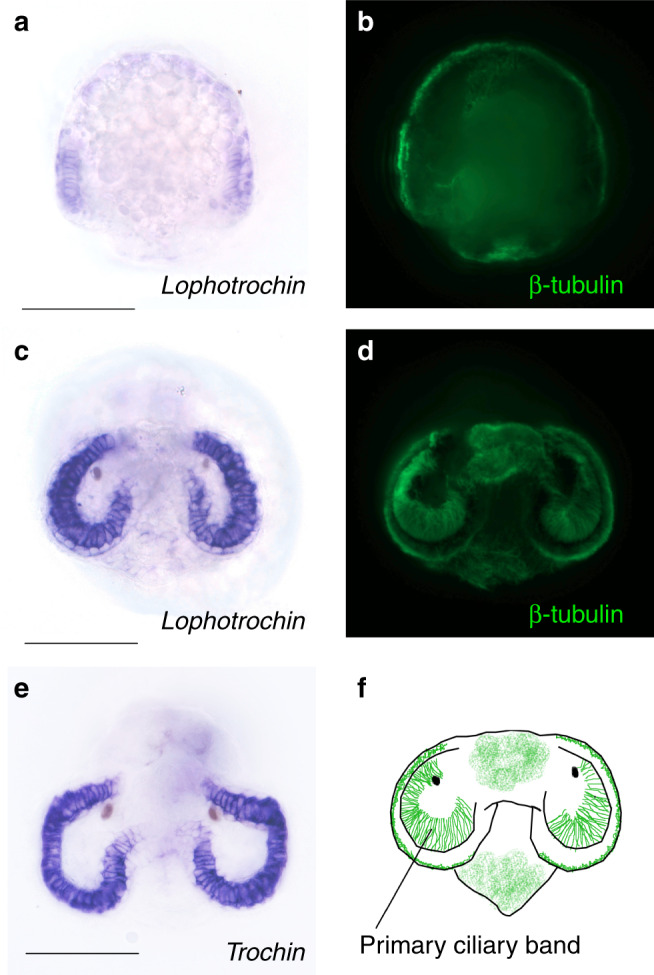
*Lophotrochin* and *trochin* expression in the mollusc *Tritia obsoleta*. **a** Anterior view of an early (around 3-day old) embryo at the onset of primary ciliary band formation, stained for *lophotrochin* (purple), and **b** for cilia (green). **c** In a later stage larva (around 7 day old), *lophotrochin* expression is specific to the primiary ciliary band (prototroch). **d** Same larva as on (**c**) showing cilia. **e** Seven-day-old larva stained for *trochin* (purple); the expression is specific to the cells of the primary ciliary band. *Lophotrochin* and *Trochin* are expressed in the ciliated cells of the midgut in later larvae (not shown). **f** Cartoon of the anterior view of larva; the primary ciliary band (green) spans the velar lobes (based on (**d**)). Other shorter cilia are found on the middle of the head, and the foot. Scale bars: 100 μm. For each in situ hybridization, at least 20 animals were stained and all had the pattern shown.

We found 37 genes that fit our criteria (Supplementary Data 1), and examined the expression of 20 of these genes at multiple stages during embryonic and larval development of the gastropod *Tritia obsoleta*, formerly known as *Ilyanassa obsoleta*^35^. Surprisingly, two genes with no sequence similarity to each other were both specifically expressed in the cells of the primary ciliary band of the larva, the prototroch, but not in other ciliary structures such as the fields of ciliated cells on the foot and on the middle of the head (the apical plate). Both genes had nearly identical expression patterns and their expression was not observed until early organogenesis when the cilia of the bands start to appear (Fig. 2). Based on their expression patterns, we named these two genes *lophotrochin* and *trochin*. To compare expression patterns of these genes with a cilia-specific gene, we performed in situ hybridization for *axonemal dynein*. Unlike *lophotrochin* and *trochin*, this transcript was detected in all ciliary structures including the fields of ciliated cells on the foot, apical plate and shell margin (Supplementary Fig. 2). Although it remains formally possible that *lophotrochin* and *trochin* are expressed below the limits of our ability to detect them in these non-ciliary band ciliated cells, the available evidence indicates that these genes are largely restricted to the ciliary bands unlike our general ciliary maker *axonemal dynein*. The other 18 genes examined were ubiquitously expressed during embryonic and larval stages.

More sensitive sequence similarity searching using PSI-BLAST revealed that the N terminal part of *Lophotrochin* has similarity to an uncharacterized domain found in some non-spiralian genes (DUF4476, pfam14771; Supplementary Fig. 1). However, the C-terminal part of the protein contains a novel motif that is specific to spiralian *lophotrochin* genes and is strongly conserved. This protein appears to have been derived from a DUF4476 domain-containing protein in the spiralian common ancestor that underwent rapid evolution to generate the new C-terminal spiralian-specific motif, or a fusion event with the novel C-terminal motif. Thus, not all of the *lophotrochin* protein sequence arose de novo in the common ancestor of spiralians, but it contains a novel sequence motif that apparently did arise in the common ancestor and was combined with an older domain. This pattern raised the possibility that *lophotrochin* might be derived from an ancestral DUF4476-containing protein that was expressed in ciliated band-like structures before the common ancestor of the spiralians. The available data does not support this. Echinoderm larvae ciliary bands are most similar to spiralian bands, but no DUF4476 domain was found in echinoderm sequences available in NCBI (see Methods).

*Trochin* has no detectable sequence similarity to any non-spiralian genes or protein domains even using multiple rounds of PSI-BLAST or using HMMER (Supplementary Fig. 3). Thus, *trochin* appears to be the result of de novo gene formation or rapid evolution in the spiralian ancestor. For both genes, the strong evolutionary constraint over the ca. 500MY since the spiralian common ancestor in the Cambrian indicates that these genes have significant functional roles.

### *Lophotrochin* and *trochin* in ciliary bands of other spiralians

To begin to understand these genes’ evolutionary history and potential function in Spiralia, we examined expression of *lophotrochin* in two polychaete annelids, a phoronid worm, a brachiopod, two nemertean worms, and a rotifer. We have less complete sampling for *trochin* (Fig. 1a).

**Fig. 3:**
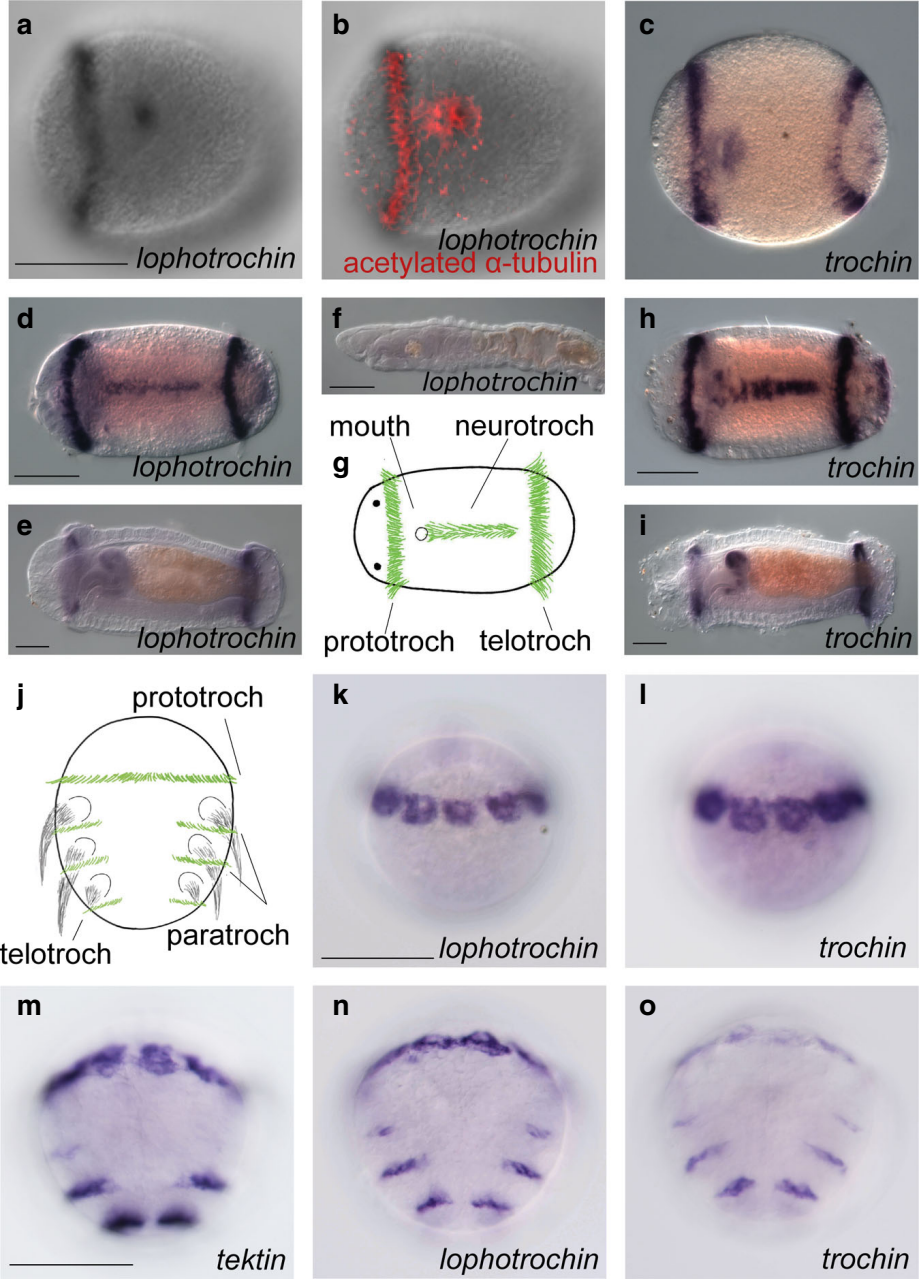
*Lophotrochin* and *trochin* expression in annelids. **a**–**i** *Capitella teleta*: *lophotrochin* and *trochin* show a similar expression pattern throughout development and expression of both genes is restricted to a subset of ciliated structures. **a** In early stage 4 larvae, *lophotrochin* (black) is expressed in the prototroch and the ciliated area around the mouth. Ventral view, brightfield. **b** Labeling of cilia in the same larva with acetylated α-tubulin antibody (red) merged with (**a**). **c** In late stage 4, *trochin* (purple) is expressed in the prototroch, the area around the mouth, and in the telotroch, ventral view. **d** *Lophotrochin* (purple) expression is seen in prototroch, telotroch and neurotroch, in stage 6, ventral view. **e** In stage 8, *lophotrochin* is expressed in the anterior esophagus and pharynx. Expression in the prototroch and telotroch is reduced compared with that in stage 6. Lateral view. **f** *Lophotrochin* expression is undetectable after metamorphosis, juvenile, lateral view. **g** Diagram of a mid-stage larva, showing the prototroch (left, vertical), neurotroch (center, horizontal), and the telotroch (right vertical band). Cilia in green. **h** *Trochin* expression in stage 6 larva, ventral view. **i** *Trochin* expression of stage 8, lateral view. **j**–**o** *Platynereis dumerilii*: expression of *lophotrochin* and *trochin* is very similar, and is restricted to ciliary bands. **j** Diagram of the ciliary bands of the larva of the annelid *Platynereis*, cilia in green and chaetae in black. **k** At 24 h post fertilization (hpf), *lophotrochin* (purple) is expressed in the prototroch cells, ventral view. **l** *Trochin*’s expression pattern (purple), 24 hpf, ventral view. **m** In 48-hpf larva, expression of a conserved ciliary marker *Tektin-3/5A* (purple) is shown in the prototroch (now a thinner ring of cells), the ciliated apical organ (out of plane in this image), a larger telotroch, and in two pairs of rows (paratroch 1, weakly, and paratroch 2) between the first three pairs of trunk segments/parapodia, ventral view. Expression of (**n**) *Lophotrochin* and (**o**) *trochin* in the same stage (48 hpf) larva show almost identical pattern to the *Tektin-3/5A* (**m**). Scale bars: 100 μm. For each in situ hybridization, at least 20 animals were stained and all had the pattern shown.

In *Capitella teleta*, a polychaete annelid with a trochophore larvae, *lophotrochin* and *trochin*, shows very similar expression patterns, and are both restricted to the main ciliary bands and a subset of other ciliated structures in the larva. Expression arises first in the prototroch, the first ciliary band to form, and expression is detectable around the time that cilia appear (Fig. 3a–c). As the cilia of the telotroch and then the neurotroch appear, both genes are expressed in these cells as well (Fig. 3d, h). Expression is also observed in a subset of other ciliated cells in the larva, including the pygidium and in the foregut (Fig. 3e, i), but the two genes are not observed in all ciliated cells. For instance, neither gene is expressed in the dorsal ciliated pad of the pharynx. Expression of both genes persists in the prototroch and telotroch through the larval period, until shortly before metamorphosis (Fig. 3e, i). The ciliary bands (prototroch, neurotroch, and telotroch) are lost during metamorphosis, and in juveniles, no expression of *lophotrochin* is detectable (Fig. 3f), while *trochin* is only expressed in the ventral face of the pharynx (Supplementary Fig. 4).

In another polychaete annelid, *Platynereis dumerilii*, *lophotrochin*, and *trochin* have similar expression patterns to *Capitella* and are expressed in the ciliary bands of the trochophore larva. The earliest expression for both genes is in the cells of the prototroch, around the stage when cilia begin to appear (Fig. 3k, l). Expression is subsequently observed in cells of several other ciliary structures: the apical tuft, the telotroch, and the paratroch bands in each developing trunk segment (Fig. 3n, o). Overall, both genes are co-expressed in all ciliated structures before and during larval growth, and persist in all multiciliated cells with long cilia. *Lophotrochin* and *trochin* have not been observed in other tissues or cell types.

**Fig. 4:**
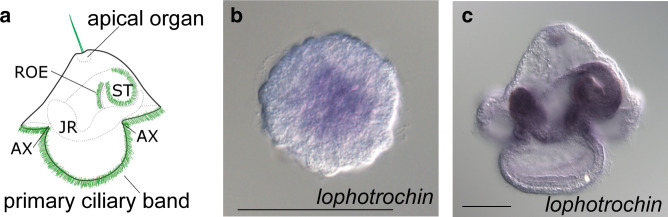
*Lophotrochin* and *trochin* expression in the nemertean *Maculaura alaskensis*. **a** Diagram of a torus-stage pilidium larva, cilia in green. Only one complement of the paired structures is shown for clarity (i.e., left lappet, left ciliary ridge, and left anterior and posterior axil). AX: axil, JR: juvenile rudiment, ROE: ciliated ridge of esophagus, ST: stomach. **b** The first expression of *lophotrochin* (purple) is noted at gastrula stage—in the developing archenteron, but not in ectodermal ciliated cells. **c** In later developmental stages, more structures with *lophotrochin* expression are found: stomach, ciliated ridges of esophagus, juvenile rudiment, the axils—recesses between lobes and lappets, the apical organ, and the primary ciliated band adjacent to the axils. Scale bars: 50 μm. For each in situ hybridization, at least 20 animals were stained and all had the pattern shown.

We examined *lophotrochin* in the nemertean worm *Maculaura alaskensis* (formerly known as *Micrura alaskensis*, see ref. ^36^), which has a typical nemertean larva, the pilidium (we did not find a *trochin* ortholog in the transcriptome data available for this species). This larva has a prominent band of multiciliated cells with which it swims and feeds (Fig. 4a); this band arises from similar cell lineages to the prototroch of annelids and molluscs, but recent studies suggest that it may be convergent: unlike the prototroch, its cells avoid the early cleavage arrest, and it functions very differently^37–39^. *Lophotrochin* is expressed in a number of structures but is not in the primary ciliated band (Fig. 4b, c). Expression is observed in the axils—the primary growth zones of the larva that generate the epidermis, including ciliary bands and the imaginal disks^40^. Expression is also noted in ciliated cells of the stomach, apical organ, and esophageal ciliary ridges. All of these possess cilia that are somewhat different in length and function to the cilia in the rest of the epidermis. Expression is additionally observed in the developing imaginal disks (Fig. 4c). So, unlike other spiralians we examined, *lophotrochin* is not expressed in the prominent ciliated band of the nemertean pilidium larvae, which is thought to be convergent with the prototroch; however, the gene is expressed in the cells that generate the bands, and otherwise is largely expressed in specialized ciliated cells compared with normal ectodermal ciliated cells.

The Spiralia also include a group of animals known as the lophophorates, named after the lophophore, a ciliated feeding organ found in adults and some larvae (Fig. 1d). Since the lophophorates are closely related to molluscs and annelids^1,2,4,6,41^, the homology of the lophophore with other spiralian ciliary bands is plausible. However, the lophophore cilia are significantly different in structure and function from those of other spiralian ciliary bands. Notably, the lophophore ciliary band cells are monociliated and collect food upstream of the beating cilia, unlike typical prototrochs, which are composed of multiciliated cells, and collect particles downstream of their ciliary band when used for feeding^14,42^. In addition, lophophores are posterior to the mouth, while prototrochs are preoral. Cell lineage data also do not support the homology of the lophophore with the prototroch. Phoronids and brachiopods do not have spiral cleavage, making it difficult at best to assign homologies between their cells and 1q^2^. In addition, fate mapping has been performed in representatives of both of these groups, and in both cases the fates of cells in the early embryo are not fixed, precluding the assignment of the lophophore fate to any particular set of cells^43–45^.

**Fig. 5:**
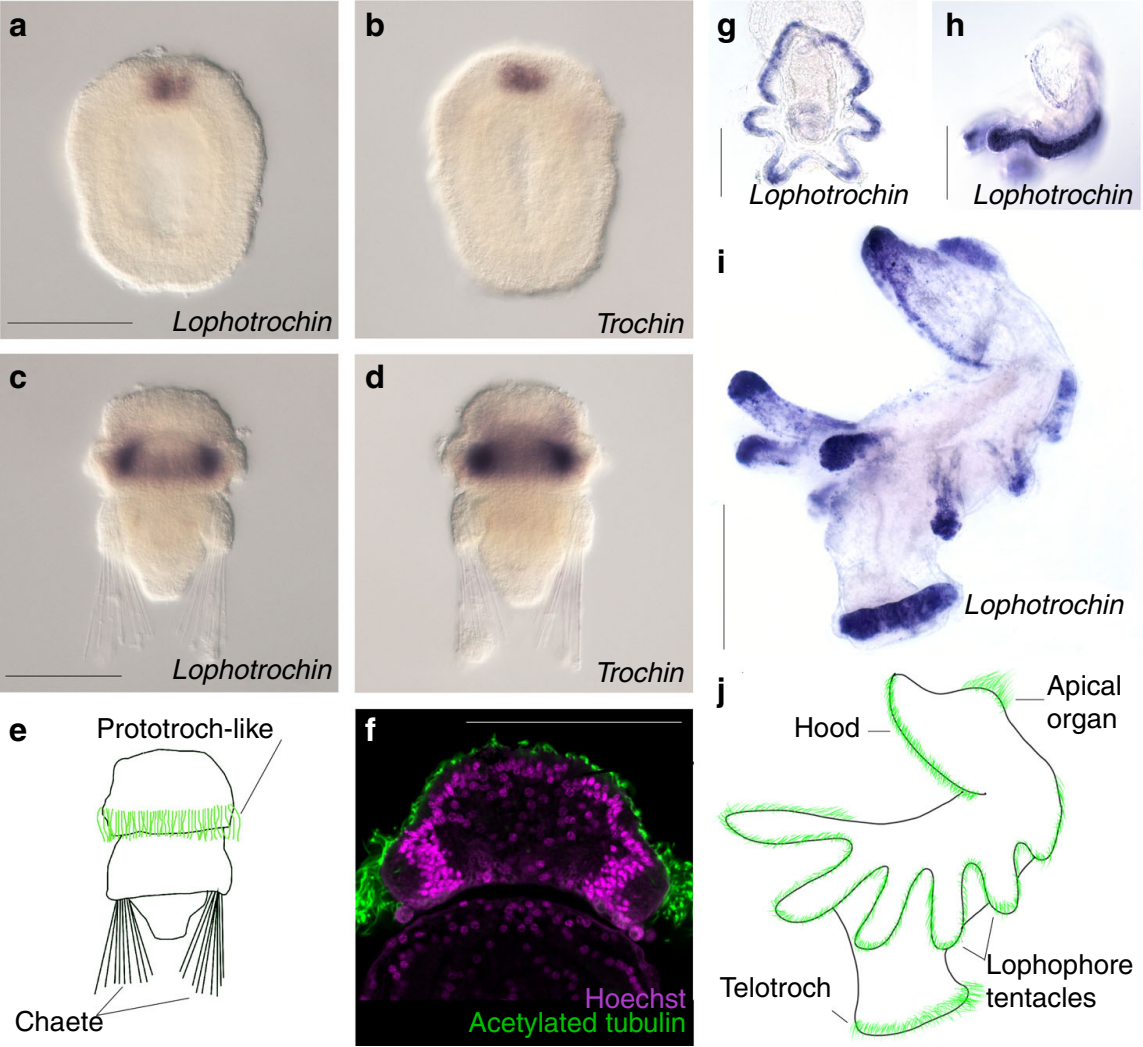
*Lophotrochin* and *trochin* expression in lophophorates: brachiopods and phoronids. **a**–**f** The *lophotrochin* and *trochin* expression in brachiopod *Terebratalia transversa*. **a** *Lophotrochin* (purple) is expressed apically in the region where the ciliary tuft forms. **b** Expression of *trochin* (purple), also in the region where ciliary tuft forms. **c** In later stage larvae, *lophotrochin* expression is specific to the ciliary band. **d** Expression of *trochin* in later larval stage is also specific to the ciliary band. **e** Diagram of the larva, based on (**c**). Cilia are highlighted in green. **f** Confocal image with nuclei (magneta, Hoechst) and cilia (green, anti-acetylated tubulin) showing that the nuclei of cells underlying the prototroch-like ciliary band are deep beneath the surface, in a similar location to the in situ probe signal. Note that the ectoderm is covered with short cilia. **g**–**j** The expression of *lophotrochin* in phoronid *Phoronopsis harmeri*. **g**, **h** In *Phoronopsi*s’s four-tentacle stage larva, *lophotrochin* expression (purple) is observed in the telotroch and along the ciliary band spanning the larval tentacles. **g** posterior view, **h** lateral view. **i** Expression becomes stronger in the tentacles as development proceeds, and in the ten-tentacle stage, expression is strongest in the tips of the largest tentacles, and the telotroch, expression is also found in the ciliated cells at the edge of the preoral lobe (or hood) and in the apical organ. **j** Diagram of the actinotroch larva showing ciliary structures (apical organ, edge of the preoral lobe, the main ciliary band spanning lophophore tentacles, and telotroch) highlighted in green. Scale bars: 100 μm. For each in situ hybridization, at least 20 animals were stained and all had the pattern shown.

We examined two lophophorates: the brachiopod *Terebratalia transversa*, and the phoronid *Phoronopsis harmeri*. In *Terebratalia*, both *lophotrochin* and *trochin* are first expressed in the apical organ (Fig. 5a, b), and in later larvae, expression is observed in the cells of the main prototroch-like circumferential ciliary band (Fig. 5c, d). Neither *lophotrochin* nor *trochin* expression is found in other ciliary structures in the larva, for instance, the ciliated cells of the apical lobe (Fig. 5f). During brachiopod metamorphosis, the adult lophophore forms from the apical lobe, in the approximate location of the prototroch-like ciliary band^46^; however, it is not known if the lophophore forms directly from these band cells.

In phoronids like *Phoronopsis harmeri*, the lophophore tentacles develop during larval stages^47^, allowing us to determine whether *lophotrochin* is expressed in the developing lophophore by examining expression patterns of larvae. In early larvae, *lophotrochin* expression is observed in the posterior ciliated band (telotroch) and the developing tentacles of the lophophore (Fig. 5g, h). Expression becomes stronger in the tentacles as development proceeds. By the ten-tentacle stage, expression is strongest in the tips of the largest tentacles and the telotroch, and is also found in the ciliated cells at the edge of the preoral lobe (or hood) and in the apical sense organ (Fig. 5i). We do see scattered positive cells in the ectoderm outside of the ciliary bands, but the entire larval epidermis is covered with cilia^48^, while the *lophotrochin* expression is strongly enriched on the tentacles and cells of the ciliary bands. These results show that *lophotrochin* and *trochin* are found in the main larval ciliary bands of brachiopod larva, and *lophotrochin* is expressed in the developing adult lophophore tentacles of a phoronid larva.

Current phylogenies indicate that the clade Gnathifera, which includes the rotifers, is an outgroup to the clade including the taxa we have examined thus far^1–3,5,6,41^. Many rotifers, like bdelloids and monogononts, are small animals that swim and feed with a prominent band of cilia around the head called the corona. Similar to the prototroch, the corona is composed of multiciliated cells, and collects food downstream of the ciliary band^14,49^. The overt similarity of the corona of some groups of rotifers to the prototroch of the trochophore larva is striking, and their homology has been proposed, but also disputed^23,50^. It is not known what cells generate the corona in any rotifer; even if it were, their embryos do not have canonical spiral cleavage, confounding any comparison between their cells and 1q^2^ in molluscs, annelids and nemerteans (reviewed in ref. ^14^).

**Fig. 6:**
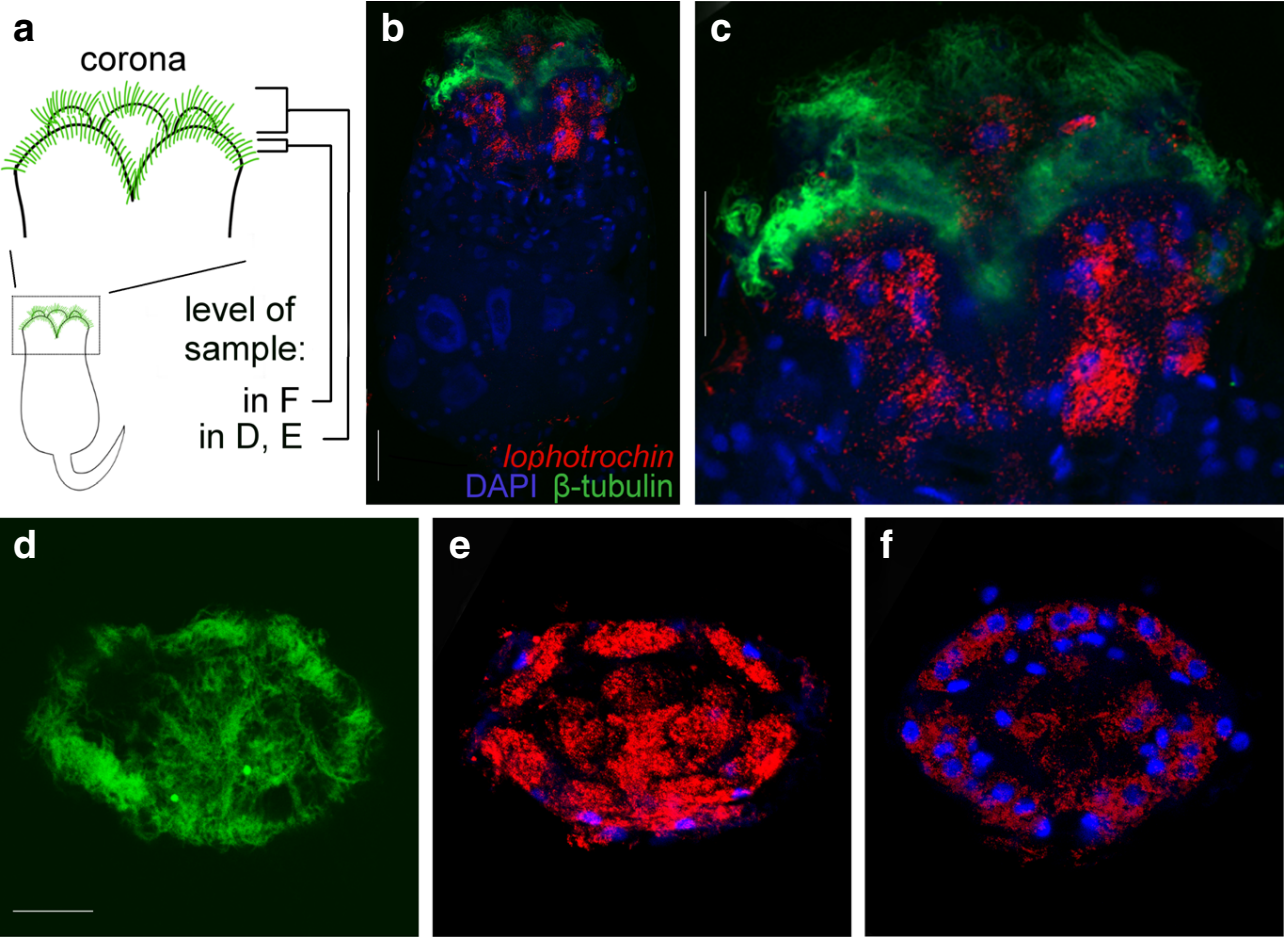
*Lophotrochin* expression in rotifer *Brachionus calyciflorus*. **a** Diagram of the *Brachionus* body plan and ciliary band (in green). Corona region enlarged. The positions of the confocal slabs of (**d**–**f**) are indicated, ventral view. **b** Confocal image, ventral view of animal, tail omitted. *Lophotrochin* is expressed in cells of the corona. Cilia in green, *lophotrochin* RNA in red, and DNA (DAPI) in blue. **c** Corona region enlarged from (**b**). **d** Anterior view of corona cilia. There are two concentric ciliary crowns in the corona: the outer crown (cingulum) and the inner crown (pseudotrochus). **e** Anterior view of *lophotrochin* RNA expression in ciliated cells. Expression can be seen in both outer and inner ciliary crowns. **f** Confocal section, posterior of (**e**). **d**–**f** are from the same individual. Scale bars: 50 μm. For each in situ hybridization, at least 20 animals were stained and all had the pattern shown.

We found *lophotrochin*, but not *trochin*, in available transcriptome data from the monogonont rotifer *Brachionus calyciflorus*, and examined expression in this animal. *Lophotrochin* is specifically expressed in the cells around the anterior that bear the corona cilia (Fig. 6). In the stages we examined, we did not detect *lophotrochin* expression in other cells bearing cilia: for example, the two ventral-lateral antennae which include a cluster of cilia^51^.

## Discussion

In all but one of the spiralians we examined, *lophotrochin* is mainly specific to ciliary band cells. In a few cases, there is also expression in ciliated gut cells and in the brachiopod and the phoronid, expression is also observed in the ciliated cells of the apical tuft. Broadly speaking, the gene is restricted to groups of cells with organized ciliary arrays (please note, we are inferring this for the ciliated gut cells). However, the strongest commonality in the expression patterns across the group is the expression in ciliated bands, which is observed all taxa except the nemertean. One possibility is that in the common ancestor of spiralians, these genes were expressed in a structure other than a ciliated band, like the apical tuft cells, and then were recruited to ciliary bands independently during the evolution of multiple spiralian lineages. Another, perhaps simpler, explanation of this pattern is that in the common ancestor of the spiralians, *lophotrochin* and perhaps *trochin* were expressed in a ciliary band, and possibly other multicellular ciliated structures like an apical organ and gut epithelium. The pattern we observe today would then have resulted from conservation of the role in ciliary bands, as well as a few cases of cooption of expression into structures other than the ciliary band, or loss from those structures. In the nemertean larva—whose main larval ciliary band is thought to be secondarily derived—*lophotrochin* is expressed in the gut, and in stem cell niches that generate the ciliary bands. This is a particularly dramatic difference, but one that may be functionally similar to other taxa even if the mRNA is not specific to the whole ciliary bands.

Since cooption of gene expression between different regions is common in evolution, shared expression of 1–2 genes in observed expression domains can only provide limited evidence for their homology. However, the distribution of ciliary bands across the Spiralia provides independent strong evidence that the common ancestor had at least one prominent ciliary band of some kind. When the phylogenetic affinity of molluscs and annelids with lophophorates was first recognized using molecular phylogenetics, the name proposed for the clade was Lophotrochozoa, combining lophophore and trochophore^4^. In our view, this name simultaneously acknowledged that there was no clear morphological synapomorphy for the clade, but hinted that the prominent ciliary bands may be a commonality. As additional taxa have been found to reside within this group, it is striking that essentially all have prominent ciliary bands at some point in their life cycle. Notably, the clade Gnathifera—which seems to be basally branching in recent spiralian phylogenies—includes phyla like chaetognaths and rotifers with prominent ciliary bands (see, e.g., ref. ^52^). In Ecdysozoa, which is the sister clade to Spiralia, ciliary bands are not observed. While this pattern indicates that the common ancestor of the Spiralia likely had one or more ciliary bands, the diversity observed among spiralian ciliary bands in their structure, function and developmental origin belies any simple reconstruction of ciliary band evolution from a common origin in the group (see Fig. 1). This could be construed as evidence of convergent origins of ciliary bands across the group; alternatively, it may reflect the age of this character, the diversity of larval and adult body plans in the Spiralia, and the diversity of roles for ciliary bands across the clade. For now, given the diversity of the ciliated bands in spiralians, the most precise definition of this putatively homologous character would be a linear array of cells, whose cilia move in a coordinated way to produce water flow for feeding and/or locomotion. It remains to be seen what additional molecular, functional or structural traits might also distinguish it.

Whether spiralian ciliary bands are derived from one that was in the common ancestor or derived independently in different lineages, the wide distribution and importance of ciliary bands in this group make them a striking and distinctive aspect of spiralian biology. This work provides an example of protein sequences that are specific to a multiphylum assemblage and associated with a putative synapomorphy of that group. It is similar to the finding that several cnidarian-specific genes with cnidarian-specific protein motifs are specifically expressed in a cnidiaran-specific cell type, the cnidocyte^25,28^. These studies and ours indicate that lineage-specific genes or protein motifs can be a rich source of genetic material for evolutionary innovation, and thus deserve further scrutiny. We speculate that ancient lineage-specific genes or protein motifs may be particularly likely to be associated with ancient synapomorphies, because they arose, or rapidly evolved at approximately the same time that the traits appeared.

## Methods

### Bioinformatic screen

We used the genome of the oyster *Crassostrea gigas* to define our starting set of potential spiralian-specific genes, because it has the most extensive transcriptome dataset of any spiralian genome, and the largest number of predicted genes^53^. We chose other spiralian and outgroup genomes based on the quality of assembly and annotation, as well as phylogenetic sampling.

Of the 11 spiralian phyla, available genomes when this analysis was performed fall into these four phyla: molluscs, annelids, rotifers and flatworms. We did not include a rotifer genome in this study because (1) only one genome in this phylum is available and (2) this genome has been shown to have high rates of lineage-specific gene loss—16% of conserved metazoan gene families have been lost^54^. So, to represent spiralian genomes we used two molluscs, *Crassostrea gigas* (oyster) and *Lottia gigantea* (limpet snail); two annelids *Capitella teleta* (polychaete worm) and *Helobdella robusta* (leech); and four flatworms: *Schistosoma mansonia* (trematode), *Schmidtea mediterranea* (planarian), *Echinococcus multilocularis* (tapeworm), and *Hymenolepis microstoma* (tapeworm). For outgroups to the Spiralia, we used the genomes of *Caenorhabditis elegans*, *Drosophila melanogaster*, *Mus musculus*, *Xenopus laevis*, *Apis mellifera*, *Ciona intestinalis*, *Hydra magnipapillata*, *Arabidopsis thaliana*, *Dictyostelium discoideum*, and *Monosiga brevicollis*.

Using in-house developed python scripts [https://github.com/longjunwu/spiralian-specific-genes], we screened for *Crassostrea gigas* genes that were detectable at a BLASTp e value below 10e−7 in at least one genome per spiralian phyla, but not present in any non-spiralian outgroup genome, or the NCBI nr database (manually searched on NCBI website) at an e value below 10e−5. We also performed a round of tBLASTn searches with an e-value cut-of 10e−7 on the spiralian genomes to find genes that may have escaped annotation.

To look for genes containing DUF4476 domain in echinoderms, on NCBI website, we used *C. gigas lophotrochin*’s (1) full protein sequence and (2) DUF4476 protein domain sequence, respectively, as search query (three iteration of PSI-BLAST, default E-value threshold) against (1) *Strongylocentrotus purpuratus* genome and (2) all echinoderm sequences in nr protein database. To look for *trochin* outside spiralians, *C. gigas trochin* was searched against NCBI nr protein database using the same PSI-BLAST method. *C. gigas trochin* was also searched against Reference Proteomes, UniProtKB, SwissProt and Pfam databases using HMMER on EMBL-EBI (ebi.ac.uk/Tools/hmmer/) with default E-value threshold.

### *Tritia obsoleta*

*Tritia obsoleta* were obtained from the Marine Resources Center at the Marine Biological Labs, Woods Hole, Massachusetts. Animals were maintained in the lab with rearing temperature 22 °C (+/−1) and were fed with frozen clams^55^. *Lophotrochin* and *trochin* gene sequences were discovered by BLAST searches against *Tritia* transcriptome database^56^. DNA templates for probes synthesis were generated via PCR from *T. obsoleta* cDNA libraries. Digoxigenin (DIG)-labeled probes were synthesized using T3 RNA polymerase and DIG-UTP from probe templates. Probes were checked by agarose gels. We used our established in situ hybridization protocol^57^. Embryo were fixed in PEM fixation buffer (10 mM EGTA, 100 mM PIPES (pH 6.9) and 1 mM MgSO_4_) for 1–2 h. Embryos were pretreated for 10 min in 10 μl ml^−1^ acetic anhydride in triethanolamine (TEA) buffer and then 10 min in 20 μl ml^−1^ acetic anhydride in TEA buffer. Prehybridization was performed for 3 h at 68 °C in Hyb solution (1× Denharts, 5× SSC, 1% Tween-20, 100 μg ml^−1^ heparin, 50% formamide, and 100 μg mg^−1^ yeast tRNA and rRNA). Hybridization was performed with a DIG-labeled probe (1–10 ng) at 68 °C for 72 h. After overnight incubation with 1:4000 dilution of alkaline phosphatase-conjugated anti-DIG antibody, in situ signal was developed using nitro blue tetrazolium chloride/5-bromo-4-chloro-3-indolyphosphate (NBT/BCIP). For immunohistochemistry, embryos were fixed in PEM for 30 min. Mouse anti-β tubulin primary antibody E7 (AB2315513, DSHB) was used at a 1:400 dilution and secondary antibody anti-mouse Alexa fluor 488 conjugated (Molecular Probes, A32723) was used at a 1:1000 dilution. In situ hybridization and antibody staining results were imaged on a Zeiss Axioplan II microscope with an Insight color camera (Diagnostic Instruments). Helicon Focus 5.6 (Helicon Soft Ltd.) was used to merge multiple focal planes images. Images were processed with Adobe Photoshop CS6 and figures were created with Adobe Illustrator CS6 or GIMP 2.8.

### *Capitella teleta*

A colony of *Capitella teleta* was maintained in the laboratory at 19 °C in glass bowls of 10 µM filtered seawater and fed with sieved ocean mud^58,59^. Distinct larval stages can be distinguished according to a published morphological staging system^59^. In summary, the first larval stage is determined by the morphological appearance of cilia at stage 3 and the last larval stage, stage 9, is competent to metamorphose into juveniles when provided with an appropriate cue. Different larval stages were manually dissected out of the maternal brood tube, relaxed for 10 min in 1:1 0.2 µM filtered seawater:0.37 M MgCl_2_ and then fixed with 4% paraformaldehyde (diluted in filtered seawater) overnight at 4 °C. To obtain juveniles, stage 9 larvae were allowed to emerge from the brood tube and transferred into a glass bowl with mud (to act as a metamorphosis cue). After 3–5 days the juveniles were recovered and transferred into a 0.5% cornmeal agar (in filtered seawater) plate to clean themselves. Prior to fixation (see above) animals were relaxed in a 0.5% cornmeal agar plate prepared with 1:1 0.2 µM filtered seawater:0.37 M MgCl_2_. The fixing solution was removed with three short phosphate buffer saline (PBS) washes followed by a gradual methanol dehydration series. Animals were stored in 100% methanol at −20 °C.

EST clones of two spiralian-specific genes were identified by BLAST searches of *Capitella teleta* EST libraries (sequences by the Joint Genome Institute, Department of Energy, Walnut Creek, CA, USA). These clones were subsequently named *Ct-lophotrochin* (CAPF15022) and *Ct-trochin* (CAPF15237) and were recovered from glycerol stocks of a pBluescript SK phagemid (Stratagene) mixed-stage complementary library. Clone CAPF15022 (*Ct-lophotrochin*) contains a 1231 bp fragment, and clone CAPF15237 (*Ct-trochin*) contains an 876 bp fragment. The orientation of each fragment in pBluescript was determined by single digest using KpnI (*Ct-lophotrochin*) or EcoRI (*Ct-trochin*). For both genes, DIG-labeled riboprobes were generated using the SP6 MEGAscript kit (Ambion).

Whole mount in situ hybridization was performed with mixed larval and juvenile stages^60^. Riboprobes were diluted to a final concentration of 1 ng µL^−1^ and hybridized for 72 h at 65 °C. Following probe removal, multiple washes with SSC buffer, and washed down to 0.05× SSC, specimens were exposed to a 1:5000 dilution of anti-DIG antibody (Roche) overnight, and subsequently detected using NBT/BCIP. A subset of in situ samples was selected for immunohistochemistry^61^. Mouse anti-acetylated α-tubulin primary antibody (Sigma) was used at a 1:400 dilution and goat anti-mouse Alexa Flour 594 secondary antibody (Molecular Probes) at a 1:500 dilution. The nuclei of all specimens were counterstained with Hoechst 33342 (Life Technologies) in a 1:1000 dilution (in PBS) for 30 min.

Specimens were mounted in 80% glycerol (in PBS). Specimens that were subjected to in situ and immunohistochemistry were imaged with a Zeiss LSM 710 confocal microscope and analyzed with Imaris (Bitplane). Specimens which had undergone in situ hybridization were imaged using an Axioskop 2 motplus compound microscope (Zeiss, Goettingen, Germany), coupled with a SPOT FLEX digital camera (Diagnostic Instruments, Inc., SterlingHeight, MI). Multiple DIC focal planes were merged using Helicon Focus (Helicon Soft Ltd., Kharkov, Ukraine). All images were processed with Adobe Photoshop CS6 (version 13.0) and figure plates were created with Adobe Illustrator CS6 (version 13.0).

### *Platynereis dumerilii*

*Platynereis dumerilii* embryos were obtained from a culture at Iowa State University, and originated from a laboratory strain from the University of Mainz, Germany^62^. *Lophotrochin* and *trochin* sequences were obtained by tBLASTn searches of transcriptomic data^63^. Genes were cloned by PCR amplification with GoTaq (Promega) using gene specific primers for each gene. 896 and 827 bp fragments covering the open reading frames were obtained for *lophotrochin* and *trochin*, respectively. Fragments were ligated into P Gem T Easy vector (Promega) and transformed into competent cells. Sequences were confirmed by Sanger sequencing. DIG-labeled antisense RNA probes were generated using linearized plasmid DNA as templates using Sp6 RNA polymerase. For in situ hybridization^64^, embryos were collected at 24 and 48 hpf^65^ and fixed in 4% paraformaldehyde solution at 4 °C overnight then stored in 100% methanol. Embryos were permeabilized with Proteinase K at a concentration of 0.1 mg ml^−1^ for 1 min, and hybridized with RNA probe at 65 °C for at least 36 h in hybridization buffer (50% formamide, 5× SSC, 50 mg ml^−1^ Heparin, 0.25% Tween-20, 1% SDS, 0.2 mg ml^−1^ Salmon Sperm DNA). In situ was developed using NBT/BCIP^64^. Embryos were imaged with a Zeiss Axioskop 2 using a Canon Eos Rebel T3 camera.

### *Maculaura alaskensis*

Adult *Maculaura alaskensis* were collected from mudflats in Coos Bay near Charleston, OR, USA. *M. alaskensis* were either reproductive upon collection or became reproductive after being kept in the laboratory for some time^66^. Adult worms were transported to and kept at the Oregon Institute of Marine Biology in a flow-through seawater system in 150-mL glass custard dishes until dissection of gametes or spawning. Oocytes were transferred to filtered seawater (FSW) and fertilized with a dilute suspension of sperm. Fertilized eggs and embryos were kept at 12–16 °C in glass custard dishes^67^. Swimming larvae of *M. alaskensis* were transferred to gallon jars at concentrations of 0.1–1 larva ml^−1^, stirred constantly and fed *Rhodomonas lens* (10^4^ cells mL^−1^) after water changes which occurred every few days.

*Lophotrochin* contigs were retrieved by BLAST searches from a *M. alaskensis* transcriptome containing assembled transcripts from mixed stages (gastrula, young feeding pilidium, cephalic-disk stage, cerebral-organ-disk stage, head-and-trunk stage, and hood to pre-metamorphosis stages), and PCR amplified from cDNA libraries derived from gastrula, cephalic disks, and hood to pre-metamorphosis stages. The PCR product was subcloned into a pGEM-t vector with T4 ligase using the pGEM-t Vector System kit (Promega), and transformed into One Shot Top10 chemically competent *E. coli* cells (Invitrogen). For the preparation of riboprobes, the plasmid was linearized with the NotI restriction enzyme. The linearized plasmid was phenol:chloroform extracted and precipitated in ethanol. The DIG-labeled RNA probe was prepared with a DIG RNA Labeling Kit (Roche), precipitated with lithium chloride, and quantified with the Qubit RNA BR Assay Kit (Invitrogen).

For in situ hybridization, live *M. alaskensis* specimens were first relaxed using 1:1 0.37 M MgCl_2_:FSW for 10 min, followed by fixation overnight at 4 °C in 4% paraformaldehyde in FSW. All wash and incubation steps were 5–10 min, unless otherwise indicated. Fixation was followed by three times washes in 1X PBS, dehydration in methanol, and storage at −20 °C. Before staining, specimens were rehydrated in PBS then washed three times in PBS with 0.1% Tween-20 (PTw). For in situ hybridization, samples were incubated in 1% TEA in PTw with 0.3% acetic anhydride, followed by 1% TEA in PTw with 0.6% acetic anhydride, then two rinses with PTw, and refixation with 4% paraformaldehyde for 30–60 min followed by four washes in PTw. Prehybridization was at 63 °C for 4 h to overnight in hybridization buffer (50% formamide, 5X saline-sodium citrate (SSC) pH 7, 50 μg mL^−1^ heparin, 0.1% Tween-20, 1% SDS, 50 μg mL^−1^ boiled salmon sperm DNA in diethyl-pyrocarbonate-treated water). The RNA probe was denatured at 80–90 °C for 10 min then added to fresh hybridization buffer at 1 ng μL^−1^ and hybridized with the samples at 63 °C for 2–3 days. The probe was washed out with hybridization buffer, followed by a graded series washes in hybridization buffer/2X SSC (75/25, 50/50, 25/75, 100/0%), followed by two washes in 0.05× SSC for 30 min at 63 °C. Next, specimens were transferred through a graded series washes in 0.05× SSC/Tris-buffered saline and Tween (TBST; 0.15 M NaCl; 0.2 M Tris buffer, pH 7.5) The wash steps were 70/30, 30/70, and 0/100%. Animals were then washed four times in TBST, and blocked in TBST with 0.1% Tween-20, 5% normal goat serum, and 2 ng μL^−1^ bovine serum albumin. Detection took place in the dark in 1X Detection Buffer (Roche) with 4.4 μL of 75 mg mL^−1^ NBT (Sigma) and 3.3 μL of 50 mg mL^−1^ BCIP (Sigma), then terminated by washing larvae in PTw. Stained specimens were mounted on slides in 80% glycerol in PBS, and examined and documented on an Olympus BX51 microscope equipped with differential interference contrast optics and a Leica DFC400 digital color camera.

### *Terebratalia transversa*

*Terebratalia transversa* were collected in San Juan Channel, between San Juan Island and Shaw Island, WA, USA. *Lophotrochin* and *trochin* were identified by BLAST searches of a *T. transversa* transcriptome database^68^. PCR amplification using primers designed from contigs of interest was performed on cDNA from mixed-stage embryonic RNA with the Advantage RT-for-PCR Kit (Clontech Laboratories, Inc., Mountain View, CA, USA). Products of the predicted size were cloned into pGEM-T vector (Promega, Madison, WI, USA) and confirmed by Sanger sequencing. Antisense RNA probes were synthesized using the DIG RNA Labeling Kit (Roche Applied Science, Mannheim, Germany), following the manufacturer’s protocol. Embryos were digested with Proteinase K (0.01 mg ml^−1^ in PTw) for 10 min, then hybridized at 62 °C for 48 h with a probe concentration of 1 ng μL^−1^. After washing, embryos were labeled with alkaline phosphatase-conjugated anti-DIG antibody and detected by staining with NBT and BCIP. The progress of staining was monitored on a stereomicroscope until they reached the desired intensity, then samples were cleared and mounted for imaging in 80% glycerol. Embryos were examined and documented on a Zeiss AxioSkop microscope equipped with Plan-Apochromat 20×/08 N.A. objective, differential interference contrast optics (Carl Zeiss, Jena, Germany) and a Zeiss AxioCam HRc digital camera with Zeiss AxioVision v4.8 software (Carl Zeiss, Jena, Germany).

Immunohistochemistry for *T. transversa* cilia was performed by labeling with anti-acetylated tubulin (T-6793, Sigma-Aldrich, St. Louis, MO, USA). Fixed embryos were rinsed three times in PBS with 0.1% Triton-X 100 (PTx). Embryos were pre-blocked in PTx with 5% normal goat serum, and incubated for 24 h in anti-acetylated tubulin at 1:500. Embryos were rinsed three times in PTx, and stained with Alexa 647 conjugated anti-mouse antibody (Molecular Probes). Nuclei were counterstained with Hoechst 33342 (Life Technologies) in a 1:1000 dilution in PBS. Stained embryos were cleared and mounted in 85% glycerol. Immunohistochemistry was visualized using a Zeiss 710 confocal microscope and data were analyzed with Zen software (Zeiss, Goettingen, Germany).

### *Phoronopsis harmeri*

*Phoronopsis harmeri* were collected at Bodega Bay, California. *Lophotrochin* was discovered by BLAST searches of *a P. harmeri* transcriptome. In situ hybridization procedures were adapted from a *T. transversa* protocol as described above^68^ but without the proteinase-K treatment step to improve tissue integrity. Imaging and image processing were performed as for *Tritia* (described above).

### *Brachionus calyciflorus*

*Brachionus calyciflorus* resting eggs were obtained from Florida Aqua Farms Inc., Florida, USA. Animals were maintained^69^ in the lab with rearing temperature 22 °C (+/−1) and were fed with green algae *Nannochloropsis gaditana. Lophotrochin* was discovered by BLAST searches of a *B. calyciflorus* transcriptome database^70^. In situ hybridization procedures were adapted from a protocol^69^. *B. calyciflorus* were fixed with 4% formaldehyde with 4% acetic acid for 15 min, then treated with 20 μl ml^−1^ acetic anhydride in 0.1 M TEA buffer for 10 min then 30 μl ml^−1^ acetic anhydride in 0.1 M TEA buffer for 10 min before the hybridization step. Without this step we saw consistent non-specific probe binding to ovaries. *B. calyciflorus* were transferred to hybridization buffer and incubated at 52 °C for 2–3 h. Hybridization buffer was removed and *B. calyciflorus* were then hybridized with DIG-labeled RNA probe at 56 °C overnight. After incubation with 1:4000 dilution of alkaline phosphatase-conjugated anti-DIG antibody overnight, in situ was developed using NBT/BCIP. Mouse anti-β tubulin primary antibody E7 (AB2315513, DSHB) staining was performed as for *Tritia* (descripted above). Imaging of the in situ hybridization staining was adapted from a reflection microscopy protocol for NBT/BCIP precipitate^71^. On a Leica SP5 confocal microscope with an accousto-optical tunable filter, we illuminated with the 405, 543, 594, and the 633-nm lasers, and collected reflected light from the 633 wavelengths. The slab projections were generated in ImageJ. Imaging and image processing were performed as for *Tritia* (descripted above).

### GenBank accession numbers for sequences used in this study

*Lophotrochin*: MT127427, MT127430, MT127432, MT127433, MT127435.

*Trochin*: MT127428, MT127431, MT127434.

*Axonemal dynein*: MT127429.

*Capitella teleta lophotrochin*: ELU14756.17^71^, *trochin*: ELT92671.1.

*Brachionus calyciflorus lophotrochin*: GACQ01014466.1 (contig19114_2335_3565)^72^.

### Reporting summary

Further information on research design is available in the Nature Research Reporting Summary linked to this article.

## Supplementary information

Supplementary information_new

Supplemental Data 1

Description of Additional Supplementary Files

Reporting Summary

## Data Availability

The authors declare that all data supporting the findings of this study are available within the article and its supplementary information files or from the corresponding author upon reasonable request. Gene sequences that support the findings of this study have been deposited in GenBank with the accession codes MT127427, MT127430, MT127432, MT127433, MT127435, MT127428, MT127431, MT127434, and MT127429.

## Code availability

The python scripts that support the findings of this study are available at https://github.com/longjunwu/spiralian-specific-genes.
