# Supplementary material for: Genes with spiralian-specific protein motifs are expressed in spiralian ciliary bands
Source: Nat Commun. 2020 Aug 20;11:4171. doi: 10.1038/s41467-020-17780-7 (PMC7441323; doi:10.1038/s41467-020-17780-7)
Supplement: Supplementary file 1 — Supplementary information_new [file 41467_2020_17780_MOESM1_ESM.pdf]

Supplementary information for

Genes with spiralian-specific protein motifs are expressed in spiralian ciliary bands

Wu et al.

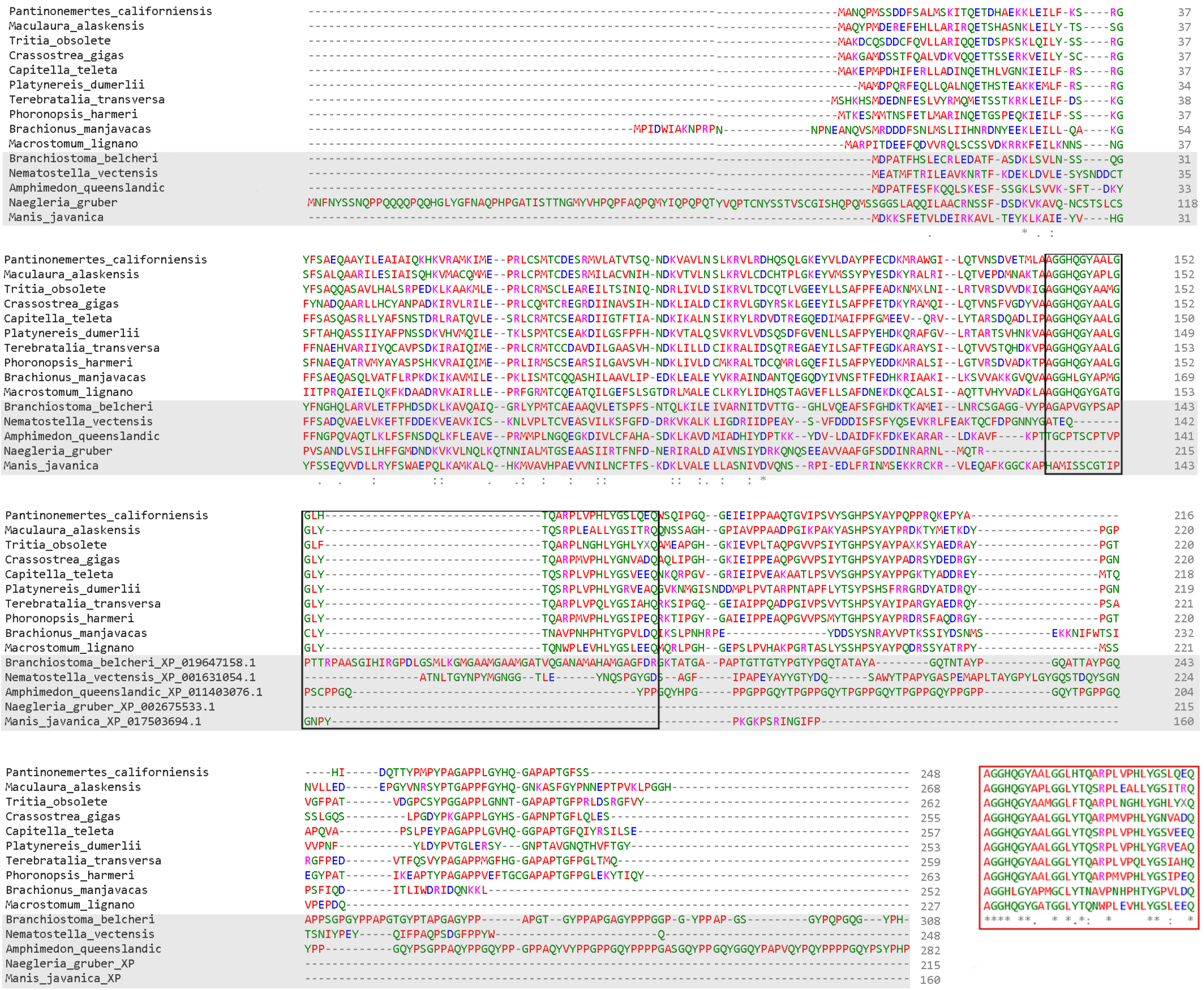

Supplementary Figure 1. Amino acid alignment of *lophotrochin*. Alignment of spiralian *lophotrochin* protein sequences and the non-spiralian sequences with highest similarity (in the grey box). The characteristic *lophotrochin* motif is highlighted in the black box and the alignment of only the spiralian *lophotrochin* motifs is separately shown in the bottom right corner in the red box.

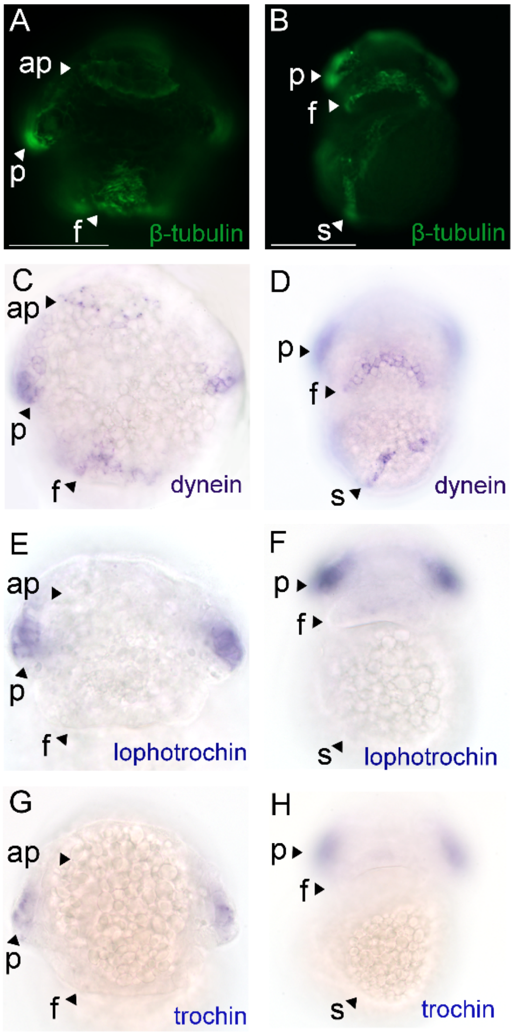

Supplementary Figure 2. *Axonemal dynein*, *lophotrochin* and *trochin* expression in the mollusc *Tritia*. A, C, E, G are the same stage early larvae, and B, D, F, H are the same stage late larvae. A, C, E, G are anterior-ventral view and B, D, F, H are ventral view. (A, B): β-tubulin staining (green) of all ciliary structures. (A): β-tubulin staining showing cilia in the apical plate, primary ciliary band and foot. (B) β-tubulin staining show ing cilia in primary ciliary band, foot and shell margin. (C, D) The *dynein* expression (purple). (C) *Dynein* is expressed in all of the ciliary structures, including the apical plate, primary ciliary band and foot. (D) *Dynein* is expressed in the apical plate, foot and shell margin. (E, F) *Lophotrochin* expression (purple) is strong in the primary ciliary band, but not detected in the other ciliary structures: apical plate, foot and shell margin. (G, H) *Trochin* expression (purple) is strong in the primary ciliary band, but not detected in the other ciliary structures: apical plate, foot and shell margin. (A, C, E, F) the imaging focus was on the level of the apical plate and foot. (B, D, F, D) the imaging focus was on the level of foot and shell margin. Abbreviations: ap: apical plate, p: primary ciliary band, f: foot, s: shell margin. Scale bar: 100 μm. For each *in situ* hybridization, at least 20 animals were stained and all had the pattern shown.

```
Capitella_teleta              MATYSFEHVNRTKANHINADTRNLLDGKQRAYPNGNYASGDGKSRVWS--GHGFYSPSAR      58
Platynereis_dumerlii          MATYTFEHINRRKATHVNADTRNLLDGRQRAYPNDNYLSGDGKSMVWM--GERYYNPKNR      58
Terebratalia_transversa       MAQYFHERLSG------------RTIKPYYPTPTNNYLSGDGKSAVWK--GHSFYVPSER      46
Phoronis_australis            -MQYYHERLSG------------RIMKPPRIYPTDNYLSGDGKSTVWPTRGSNFYVPSER      47
Macrostomum_lignano           -M----AARNCYSKYST----DPAFTGPKIVTPPNNYLSGNGKSEVYQ--GAGWYVPSER      49
Notospermus_geniculatus       MM----AARDCYSKYSL----DKRFSGPKIITPTHNYLSGDWKSFCYE--RSSYYTPSER      50
Crassostrea_gigas             MH---------RPEYGTSAGTRLFDRGPYNSTPVDNYPSGDGKSMIWR--GPSHYVPSER      49
Tritia_obsoleta               MQ----TGTTRTSSYNI--KEDLL--KRNHSTPSNNYFSGDGKSFVWS--GAAYYVPSER      50
                                                              *  ** **: **  :       * *. *

Capitella_teleta              KWQKLHEVKALPNDRIPDLIEFQSEDDWRKWQNRRDRVD-SKNCAGSGFRNACPPLLKLD      117
Platynereis_dumerlii          DWMKHHDVKGAPNARVPGQIEFPHEDAWREWQKNRDTPGSGSVCASSGFRYSAKPELKLD      118
Terebratalia_transversa       RFVKYHEYRALPPERRADAKDYQREDDWRKFQYQRDRPKG---YFVQNYKFTDTPELKLH      103
Phoronis_australis            RFIKYHDVKHLPPERRADQKEYMSEDEWRAWQAKRDAPP----YYPNGLKLTNMPPLRLD      103
Macrostomum_lignano           RWQRYHEYTALPSSSRRDAIDFQSEDDFVQFRQARDRPRA-PMLTSY-IPGEKPADLKLT      107
Notospermus_geniculatus       RWIKYHDYKHIPREARRDAIDFQSEDKWVEFQRHRDAPRG-DKLNPGGIGHSNIPELQLF      109
Crassostrea_gigas             RWTDYHQYRSLPRDTRRDAKEMQSEDQWVDFMRQRDTPSG-YYHKDIGLLQTGVPECRLF      108
Tritia_obsoleta               SWIKYPEHRSLPSDTKRDAIDMQSEDSWVRFMRNRDQPSG-YYFPRVGIRPSNVPELKLS      109
                               :    :    *     .  :   ** :  :   **                     :*

Capitella_teleta              GYAFNPFNLYRTGVPAQTLYNTHTPWPKTDARSFPSWRGPRGYYGYYHEELDIHHNGGYR      177
Platynereis_dumerlii          GYAFNPYNLYRTGTPAVTMYNPHNPWPKTDVRQFPTWRGPRGYYGYYHEELDVHHNGGYK      178
Terebratalia_transversa       GYCTNPFNLHRTGVEARTLYDP-----LPDGINNKSWRGPHGYYGYYHERLDARDTGGFR      158
Phoronis_australis            GYCMNPFNLHRTGVQARSVYNP-----KPDLI-DPTWRGPHGYYGYYHERLDSRDTKGIR      157
Macrostomum_lignano           GYRREDS-----VVPEHSQFS---TYERFPR------WQGSGYYGYYHEALDLHDGGRMK      153
Notospermus_geniculatus       GYTRDPA-----SYPAVTQYQ---PPARFPQ------WSGPGYYGYYHENLEHHASGRYR      155
Crassostrea_gigas             GYTRDLP-----SMPPRALQD--LSWPKSDAFVPLERSDRGKYYGYYHEAIARERQQRKK      161
Tritia_obsoleta               GYTRTLP-----SMPGRELFL--QPWPKCDAWTPPVRRGRGEYYGYYHERIENERRRREM      162
                              **                                        ******* :  .

Capitella_teleta              FPRDP-KALA----NDEDAMKYEYMRNQPSLHQKQP------VEFPVLL  215
Platynereis_dumerlii          FPRDP-KVLV----NDEDIYKYEYMRNVPSVHQKLP------VEFPVLQ  216
Terebratalia_transversa       IT----KQLY----NDEDYLKWEQMRTRDDPNNLLGITETARLTLPLV*  198
Phoronis_australis            LY----KQLY----NDEDWLKWEQYKTLATKT-----------------  181
Macrostomum_lignano           TLPALPRYPGYPLVSSVTGI-----------------------------  173
Notospermus_geniculatus       DLPAIRRYPGYPIMSNMTGN-----------------------------  175
Crassostrea_gigas             ELPESMRVEARDIPML---------------------------------  177
Tritia_obsoleta               EMPITQRIDASHVPTFAYNSRWTFDQP----------------------  189
```

Supplementary Figure 3. Amino acid alignment of *trochin* protein sequences.

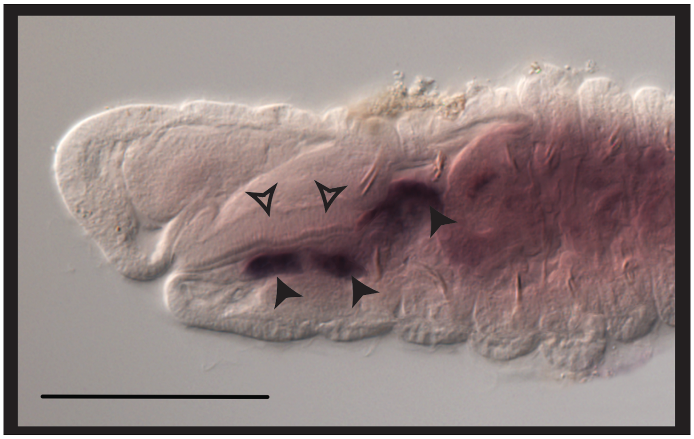

Supplementary Figure 4. Expression of *Ct-trochin* in the juvenile stage of *Capitella teleta*. After metamorphosis, expression is only observed in the ventral face of the pharynx (black arrowheads) but not the dorsal portion (open arrowheads). Scale bar: 100 um. For each *in situ* hybridization, at least 20 animals were stained and all had the pattern shown.
