# Supplementary material for: Genes with spiralian-specific protein motifs are expressed in spiralian ciliary bands
Source: Nat Commun. 2020 Aug 20;11:4171. doi: 10.1038/s41467-020-17780-7 (PMC7441323; doi:10.1038/s41467-020-17780-7)
Supplement: Supplementary file 3 — Description of Additional Supplementary Files [file 41467_2020_17780_MOESM3_ESM.pdf]

Title: Supplemental Data 1

Description: Spiralian-specific genes and their protein sequences.
