# Supplementary material for: Genes with spiralian-specific protein motifs are expressed in spiralian ciliary bands
Source: Nat Commun. 2020 Aug 20;11:4171. doi: 10.1038/s41467-020-17780-7 (PMC7441323; doi:10.1038/s41467-020-17780-7)
Supplement: Supplementary file 4 — Reporting Summary [file 41467_2020_17780_MOESM4_ESM.pdf]

natureresearch

Corresponding author(s): J. David Lambert

Last updated by author(s): 04/30/2020

Reporting Summary

Nature Research wishes to improve the reproducibility of the work that we publish. This form provides structure for consistency and transparency in reporting. For further information on Nature Research policies, seeAuthors & Referees and theEditorial Policy Checklist .

Statistics

For all statistical analyses, confirm that the following items are present in the figure legend, table legend, main text, or Methods section.

n/a	Confirmed	
☒	☐	The exact sample size (*n*) for each experimental group/condition, given as a discrete number and unit of measurement
☒	☐	A statement on whether measurements were taken from distinct samples or whether the same sample was measured repeatedly
☒	☐	The statistical test(s) used AND whether they are one- or two-sided<br>*Only common tests should be described solely by name; describe more complex techniques in the Methods section.*
☒	☐	A description of all covariates tested
☒	☐	A description of any assumptions or corrections, such as tests of normality and adjustment for multiple comparisons
☒	☐	A full description of the statistical parameters including central tendency (e.g. means) or other basic estimates (e.g. regression coefficient) AND variation (e.g. standard deviation) or associated estimates of uncertainty (e.g. confidence intervals)
☒	☐	For null hypothesis testing, the test statistic (e.g. *F*, *t*, *r*) with confidence intervals, effect sizes, degrees of freedom and *P* value noted<br>*Give P values as exact values whenever suitable.*
☒	☐	For Bayesian analysis, information on the choice of priors and Markov chain Monte Carlo settings
☒	☐	For hierarchical and complex designs, identification of the appropriate level for tests and full reporting of outcomes
☒	☐	Estimates of effect sizes (e.g. Cohen's *d*, Pearson's *r*), indicating how they were calculated

Our web collection on statistics for biologists contains articles on many of the points above.

Software and code

Policy information about availability of computer code

Data collection	The following standard programs were used for image acquisition and preparation of figures: AxioVision 4.6 (Zeiss); Spot Advanced 4 (Spot Imaging); ImageJ 1.52a; uManager; Imaris (Bitplane); Helicon focus 5.6 (Heliconsoft); GIMP 2.8; Adobe photoshop and Illustrator (CS5, CS6; Adobe).
Data analysis	As shown in Data collection.

For manuscripts utilizing custom algorithms or software that are central to the research but not yet described in published literature, software must be made available to editors/reviewers. We strongly encourage code deposition in a community repository (e.g. GitHub). See the Nature Research guidelines for submitting code & software for further information.

Data

Policy information about availability of data

All manuscripts must include a data availability statement. This statement should provide the following information, where applicable:

- Accession codes, unique identifiers, or web links for publicly available datasets
- A list of figures that have associated raw data
- A description of any restrictions on data availability

Gene sequences that support the findings of this study have been deposited in GenBank with the accession codes MT127427, MT127430, MT127432, MT127433, MT127435, MT127428, MT127431, MT127434 and MT127429.

Field-specific reporting

Please select the one below that is the best fit for your research. If you are not sure, read the appropriate sections before making your selection.

Life sciences study design

All studies must disclose on these points even when the disclosure is negative.

Sample size	No sample size calculation was performed. Many embryos of each developmental stage were included in the experiments.
Data exclusions	No data were excluded.
Replication	Many embryos of each developmental stage were included in the experiments and the expression patterns are consistent.
Randomization	Animals were randomly assigned.
Blinding	Investigators were not blinded.

Reporting for specific materials, systems and methods

We require information from authors about some types of materials, experimental systems and methods used in many studies. Here, indicate whether each material, system or method listed is relevant to your study. If you are not sure if a list item applies to your research, read the appropriate section before selecting a response.

Materials & experimental systems

n/a	Involved in the study	
☐	☒	Antibodies
☒	☐	Eukaryotic cell lines
☒	☐	Palaeontology
☐	☒	Animals and other organisms
☒	☐	Human research participants
☒	☐	Clinical data

Methods

n/a	Involved in the study	
☒	☐	ChIP-seq
☒	☐	Flow cytometry
☒	☐	MRI-based neuroimaging

Antibodies

Antibodies used

Primary antibody:
mouse anti-β tubulin (DSHB, AB2315513)
mouse anti-acetylated α-tubulin (Sigma, T7451)

Secondary antibody:
anti-mouse Alexa fluor 594 conjugated (Molecular Probes, A11005)
anti-mouse Alexa fluor 647 conjugated (Molecular Probes, A32728)
anti-mouse Alexa fluor 488 conjugated (Molecular Probes, A32723)

Validation

These antibodies are widely used in various animals, clearly stain cilia, and no special validation was performed.

Animals and other organisms

Policy information about studies involving animals; ARRIVE guidelines recommended for reporting animal research

Laboratory animals

Capitella:
A colony of Capitella teleta was maintained in the laboratory following previously published culture conditions (Grassle and Grassle 1976, Seaver et al. 2005).

Platynereis:
Platynereis dumerilii embryos were obtained from a culture at Iowa State University cared for according to published protocols available at http://www.platynereis.de (Fischer and Dorresteijn, 2004).

Brachionis:
Brachionis calyciflorus resting eggs were obtained from Florida Aqua Farms Inc., Florida. Animal maintenance was as described (Smith et al., 2010) except that we fed them green algae Nannochloropsis gaditan.

Wild animals

This study utilized marine invertebrate animals collected from the wild as indicated in the Field-collected samples section below.

Field-collected samples

Tritia:
Animals were obtained from the wild by the Marine Resources Center at the Marine Biological Labs, Woods Hole, Massachusetts,

and maintained as described (Gharbiah et al., 2009).

Maculaura:
Adult Maculaura alaskensis, were collected from mudflats in Coos Bay near Charleston, OR, USA. Larval cultures were obtained and maintained as described in (Hiebert and Maslakova, 2015).

Terebratalia:
Terebratalia transversa were collected in San Juan Channel, between San Juan Island and Shaw Island, WA, USA.

Phoronopsis:
Phoronopsis harmeri were collected at Bodega Bay, CA, USA.

Ethics oversight	No ethical approval or guidance was required because all the studied species are invertebrates.

Note that full information on the approval of the study protocol must also be provided in the manuscript.
